# A Meta-Analysis of Observational Studies on the Association of Chronic Urticaria With Symptoms of Depression and Anxiety

**DOI:** 10.3389/fmed.2020.00039

**Published:** 2020-02-27

**Authors:** Yuzhou Huang, Yi Xiao, Xingyu Zhang, Jie Li, Xiang Chen, Minxue Shen

**Affiliations:** ^1^Department of Dermatology, Xiangya Hospital, Central South University, Changsha, China; ^2^Hunan Engineering Research Center of Skin Health and Disease, Central South University, Changsha, China; ^3^Hunan Key Laboratory of Skin Cancer and Psoriasis, Central South University, Changsha, China; ^4^Department of Social Medicine and Health Management, Xiangya School of Public Health, Central South University, Changsha, China

**Keywords:** chronic urticaria, depression, anxiety, meta-analysis, observational study

## Abstract

**Background:** Chronic urticaria (CU) is a frequently occurring skin condition associated with many psychological factors, but the effect size of associations varied in literature.

**Objectives:** To perform a systematic review and meta-analysis on the associations of CU with the symptoms of depression and anxiety.

**Methods:** According to a pre-specified protocol, we systematically searched articles published in PubMed, Web of Science, CNKI, and CQVIP databases between January 2000 and January 2019. Pooled estimates in terms of odds ratios (ORs) or standardized mean differences (SMDs) were calculated according to outcome measures. Subgroup analysis by disease subtypes and tool of measurement, and sensitivity analysis were performed. Risk of bias and quality of studies were evaluated.

**Results:** Twelve studies were selected for the systematic review. The ORs were 3.99 [95% confidence interval (CI): 3.24–4.91, *P* < 0.001] for anxiety and 2.94 (95% CI: 2.42–3.58, *P* < 0.001) for depression. The SMDs of severity were 0.98 for anxiety (95% CI: 0.76–1.200, *P* < 0.001) and 0.84 for depression (95% CI: 0.59–1.10, *P* < 0.001). Subgroup analysis by disease subtypes and tool of measurement showed variations in effect size, where chronic spontaneous urticaria showed greater effects on anxiety (OR = 6.62, 95% CI: 3.67–11.95, *P* < 0.001) and depression (OR = 6.13, 95% CI: 2.31–16.31, *P* < 0.001). Sensitivity analysis demonstrated consistent results.

**Conclusion:** CU is associated with higher risks of anxiety and depression.

## Introduction

Chronic urticaria (CU) is one of the most common skin disorders characterized by the rapid appearance of wheals, angioedema, or both, for more than 6 weeks (1). The symptoms of CU are caused by the activation of skin mast cells and subsequent histamine release and other inflammatory mediators. The annual prevalence of CU is estimated to be 0.5–5% in the general population (2, 3). CU is divided into chronic spontaneous urticaria (CSU) and chronic inducible urticaria (CIndU) (1, 4). CSU occurs spontaneously with no identifiable triggers, while CIndU is characterized by the need for specific triggers (4).

It is estimated that at least 30% of patients with skin disease have significant psychiatric or psychological morbidities (5, 6). Previous studies consistently showed that anxiety and depression are common psychological comorbidities of itching-related skin diseases (7–9). Many studies have shown that patients with CU often develop psychiatric complications (10, 11). The most common diagnoses of mental disorders observed in patients with CU are depression, anxiety, and somatoform disorders (11). Patients with CSU even have more psychiatric co-morbidities than patients with psoriasis and atopic dermatitis (12). The effect of CSU on the quality of life was reported as similar to that of cardiovascular diseases (13). Psychological complications are often one of the most important indicators of overall disability associated with skin conditions.

Globally, urticaria was estimated to contribute to 4.7 million age-standardized disability-adjusted life years (14). However, the disability weights used to estimate the burden of non-communicable skin diseases are primarily determined by disfigurement and cutaneous symptoms. Psychological impacts and health-related quality of life are neglected but import aspects of skin disorders. Unfortunately, the number of people suffering from anxiety and depression in skin diseases in European countries is largely underestimated by dermatologists (15). In addition, the effect size of associations shows large variations owing to the heterogeneities in population, setting, tools of measurement, criteria of diagnosis, sample size, etc.

The current systematic review of published data aimed to assess the association of CU with the symptoms of anxiety and depression in observational studies, and to further provide a synthesis of reported effect sizes of associations. The protocol of this meta-analysis has been registered on PROSPERO (CRD42018117095), and the study is reported based on the Preferred Reporting Items for Systemic Reviews and Meta-Analysis (PRISMA) guidelines.

## Methods

### Search Strategy

We searched articles published in PubMed, Web of Science, Chinese National Knowledge Infrastructure (CNKI), and Chinese Scientific Journals Full Text Database (CQVIP), including full-text articles in both English and Chinese. The Chinese literature was searched using Chinese keywords. The searching strategy applied in PubMed is shown below:

1. Urticaria
2. Chronic urticaria
3. 1 or 2
4. Psychopathology
5. Stress
6. Depression
7. Anxiety
8. psychiatry^*^
9. Or/4–8
10. 3 and 9.

**Figure 1:**
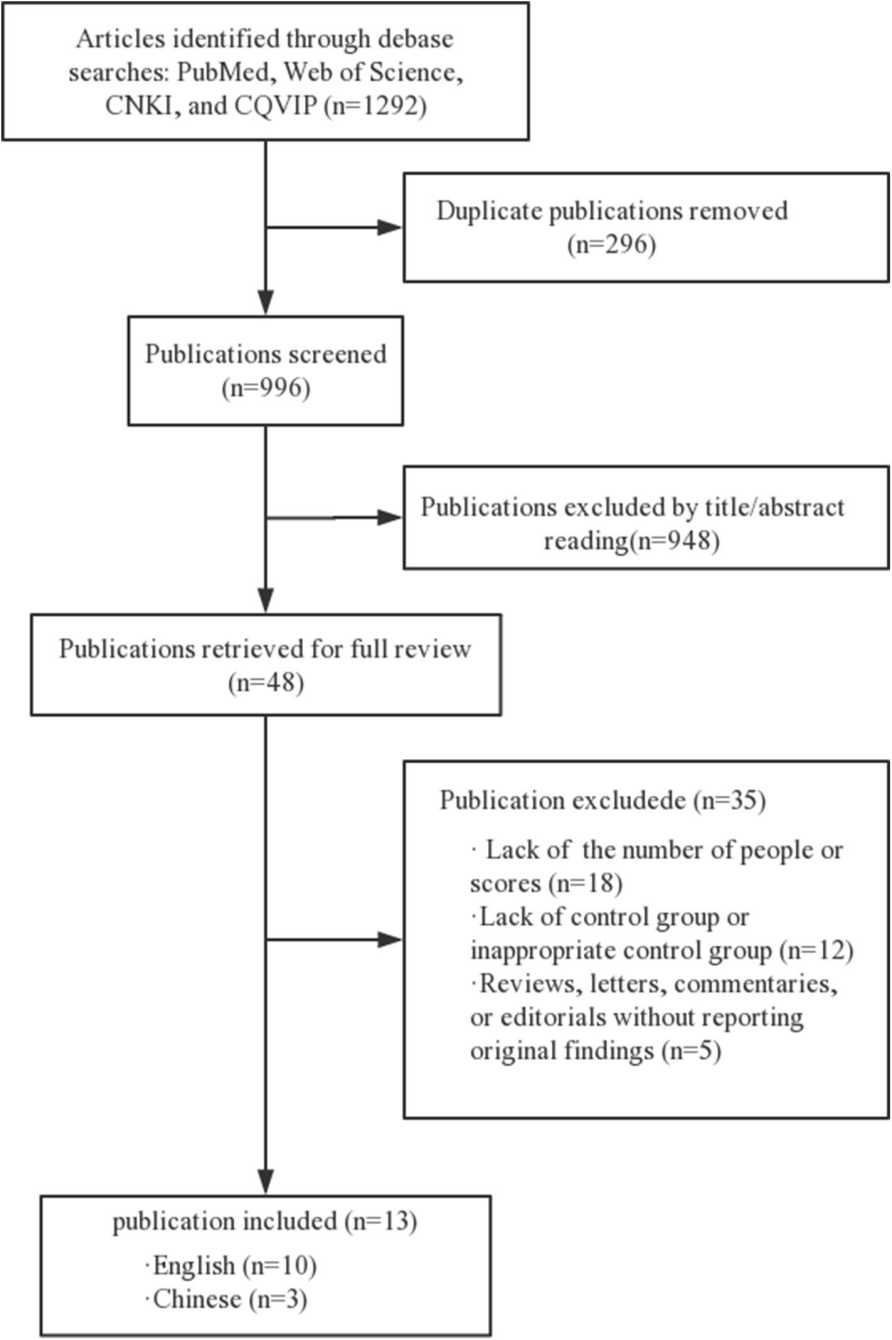
Selection process for study inclusion in the systematic review and meta-analysis.

We searched the articles between January 2000 and March 2019, in English or Chinese (Figure 1). The search strategy adjusts to a controlled vocabulary for each database. Gray literature and conference abstracts have not been searched.

### Study Selection

Studies are considered to meet the eligibility criteria if (1) hospital-based or population-based observational study, including cross-sectional study, case-control study, and cohort study; (2) there is a clear diagnosis of CU; (3) they are published in either English or Chinese; (4) there is a clear diagnosis or standardized measurement of anxiety or depression, such as a doctor's diagnosis or medical history; (5) effect size is reported as odds ratio (OR) or mean difference with 95% confidence intervals (CIs). The exclusion criteria were (1) CU is not diagnosed in detail or is measured by unvalidated tools; (2) no control group; (3) local and/or federal government reports that are not peer reviewed, as well as summaries and presentations of meetings; (4) publication in languages other than English or Chinese.

The articles were evaluated by two investigators independently using pre-specified qualification forms based on eligibility criteria and were forward looking. Any inconsistency between the investigators was resolved by consensus.

### Data Extraction

Relevant information was extracted and recorded on a data collection form (e.g., name of the first author, year of publication, country of origin, study duration, number and characteristics of participants, and identification of depression and anxiety).

### Study Quality Assessment

The quality of included observational studies was assessed using the Newcastle-Ottawa Scale (NOS) (16). The NOS includes a series of questions that are used to assess the choice of study participants, the comparability of the population, and the determination of exposure or outcomes with a maximum score of 9. Studies with scores ≥5 were classified as high-quality studies. Using these checklists, two reviewers evaluated each of the included articles for their quality. Divergent views were resolved by consultation with a third reviewer.

### Statistical Analysis

For dichotomous outcomes (anxiety and depression), the odds ratios (ORs) with 95% confidence intervals (CIs) were estimated. For continuous data (severity of symptoms), standardized mean differences (SMDs) with 95% CIs were calculated. To generate the pooled estimates of the outcomes, fixed effect models or random effect models were used according to the heterogeneity between studies. To assess the extent to which statistical heterogeneity in meta-analysis is due to differences between studies rather than accidental, we used *I*^2^ statistic and Cochran's Q (with corresponding *P*-values) (17). The forest plots were used for a visual representation of the presence and nature of statistical heterogeneity. In the subgroup analysis, subtypes of CU (including CSU, CIndU, and unspecified CU) and tools to measure (ascertain) anxiety and depression were evaluated. In sensitivity analysis, one study was eliminated at a time, and the pooled estimate was calculated. Egger's test was used to assess publication bias (18). A value of *P* < 0.05 was considered statistically significant. All statistical analyses were performed using the STATA software, version 14.0 (StataCorp, College Station, TX, USA).

## Results

### Data Sources and Selection Process

A total of 1,292 articles were selected in our screening. Of these, 296 duplicated articles were excluded and 948 articles were excluded after initial screening because they were not observational studies, or the subjects were not related to our topic. Of the full-text articles, 48 were retained for further screening. Of these, 35 were excluded for various reasons (Figure 1). Thirteen studies with a total of 3,627 study participants were included in the final meta-analysis (19–31).

### Characteristics of Included Studies

The characteristics of the included studies are summarized in Table 1. There were three cross-sectional studies and nine case-control studies. There were a total of 1,116 CU patients and 2,511 controls, with a mean age of participants ranging from 28.2 to 46.3 years, except the study in children.

**Table 1:** Characteristics of the included studies.

**References**	**Country**	**Study design**	**Participants**	**Mean age**	**Measurement/diagnosis**	
					**Anxiety**	**Depression**
Balp et al. (26)	Switzerland	Cross-sectional	369 CU patients and 1,476 health controls	44.4 vs. 44.4	Self-report	Self-report
Balp et al. (30)	Switzerland	Cross-sectional	127 CU patients and 508 health controls	37.1 vs. 37.1	Self-report	Self-report
Barbosa et al. (31)	Portugal	Case-control	55 CSU patients and 31 health controls	45.3 vs. 39.5	HADS (>7)	HADS (>7)
Brzoza et al. (21)	Poland	Case-control	54 CU patients and 59 health controls	33.0 vs. 35.0	STAI	BDI (>11)
Engin et al. (22)	Turkey	Case-control	73 patients with CSU and 34 healthy subjects	37.4 vs. 36.1	BAI (>45)	BDI (>11)
Herguner et al. (24)	Turkey	Case-control	27 children with CSU and 27 matched controls	10.5 vs. 10.7	K-SADS-PL	K-SADS-PL
Ograzyk et al. (28)	Poland	Case-control	46 female CU patients and 33 female controls	44.6 vs. 46.3	HADS (>7)	HADS (>7)
Pasaoglu et al. (20)	Turkey	Cross-sectional	59 CSU patients and 59 health controls	38.6 vs. 31.6	NA	MMPI
Tat (29)	Turkey	Case-control	50 CU patients and 60 health controls	38.3 vs. 37.1	HADS(>10)	HADS (>7)
Uguz et al. (23)	Turkey	Case-control	89 CSU patients and 64 hospital controls	36.8 vs. 32.5	Axis I and Axis II diagnoses	Axis I and Axis II diagnoses
Wu et al. (19)	China	Case-control	36 CU patients and 30 health controls	38.0 vs. 34.3	SAS (>50)	SDS (>53)
Zhang and Hou (25)	China	Case-control	31 CIndU patients and 30 health controls	31.2 vs. 33.4	N/A	SDS (>53)
Zhang et al. (27)	China	Case-control	100 CIndU patients and 100 health controls	30.2 vs. 32.5	SAS (>50)	SDS (>53)

*BAI, Beck Anxiety Inventory; BDI, Beck Depression Inventory; HADS, Hospital Anxiety and Depression Scale; K-SADS-PL, Schedule for Affective Disorders and Schizophrenia for School Age Children–Present and Lifetime Version–Turkish Version; MMPI, Minnesota Multiphasic Personality Inventory; SAS, Self-Rating Anxiety Scale; SDS, Self-rating depression scale; STAI, Trait Anxiety Inventory; CU, chronic urticaria; CSU, chronic spontaneous urticaria; CIndU, chronic-induced urticaria; N/A, not available*.

### Main Analysis

**Figure 2:**
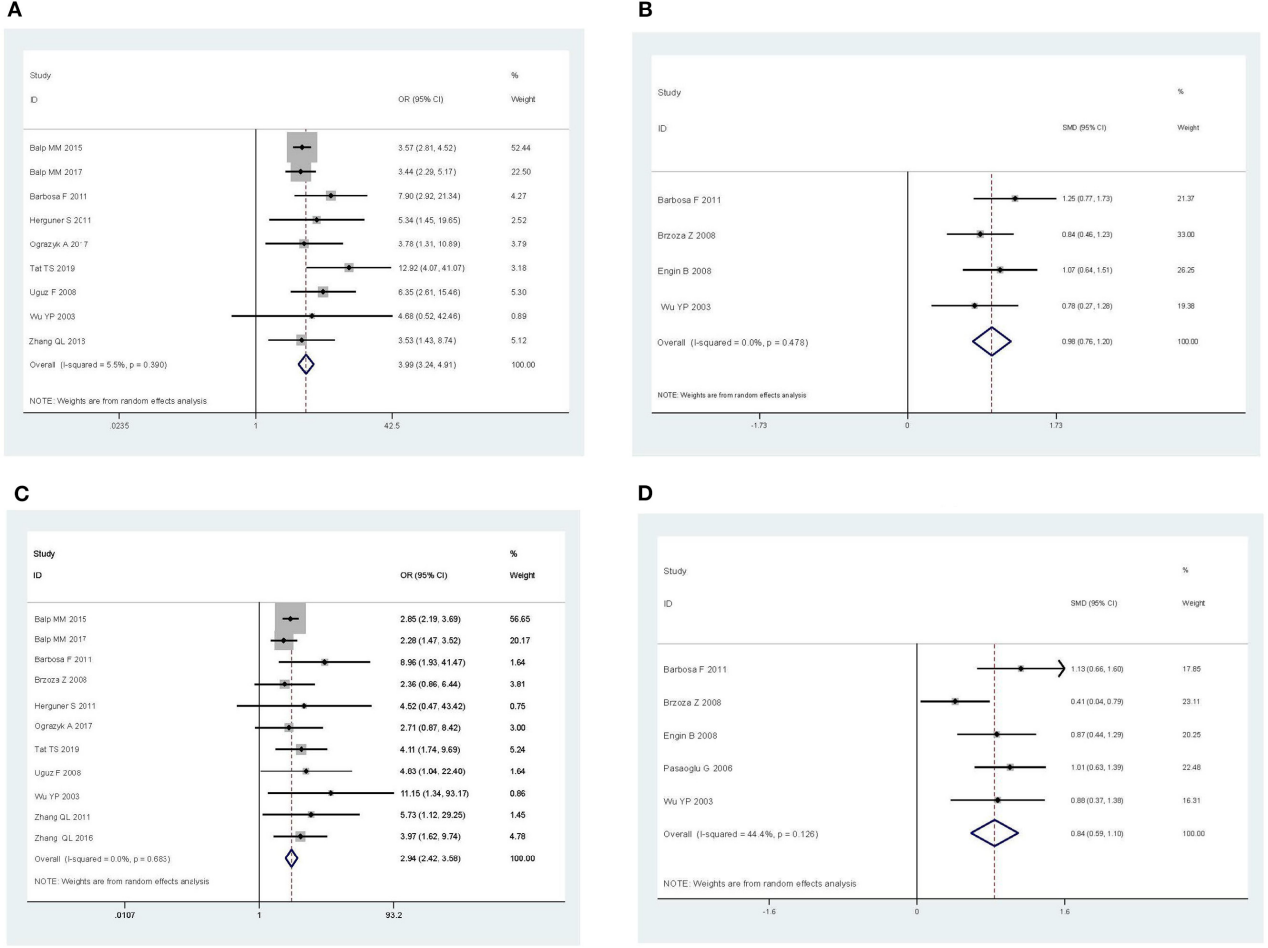
Pooled estimates of the associations of chronic urticaria with symptoms of anxiety and depression. **(A)** Anxiety, in terms of odds ratio. **(B)** Anxiety, in terms of standardized mean difference. **(C)** Depression, in terms of odds ratio. **(D)** Depression, in terms of standardized mean difference.

As shown in Figure 2, the pooled OR was 3.99 (95% CI: 3.24–4.91, *P* < 0.001) for anxiety and 2.94 (95% CI: 2.42–3.58, *P* < 0.001) for depression. If excluding the study in children, the pooled ORs were 4.08 (95% CI: 3.20–5.21, *P* < 0.001) for anxiety and 2.94 (95% CI: 2.41–3.58, *P* < 0.001) for depression. In studies that measured the symptoms of anxiety and depression as severity scores, the pooled SMDs was 0.98 for anxiety (95% CI: 0.76–1.200, *P* < 0.001) and 0.84 for depression (95% CI: 0.59–1.10, *P* < 0.001).

### Subgroup Analysis

**Figure 3:**
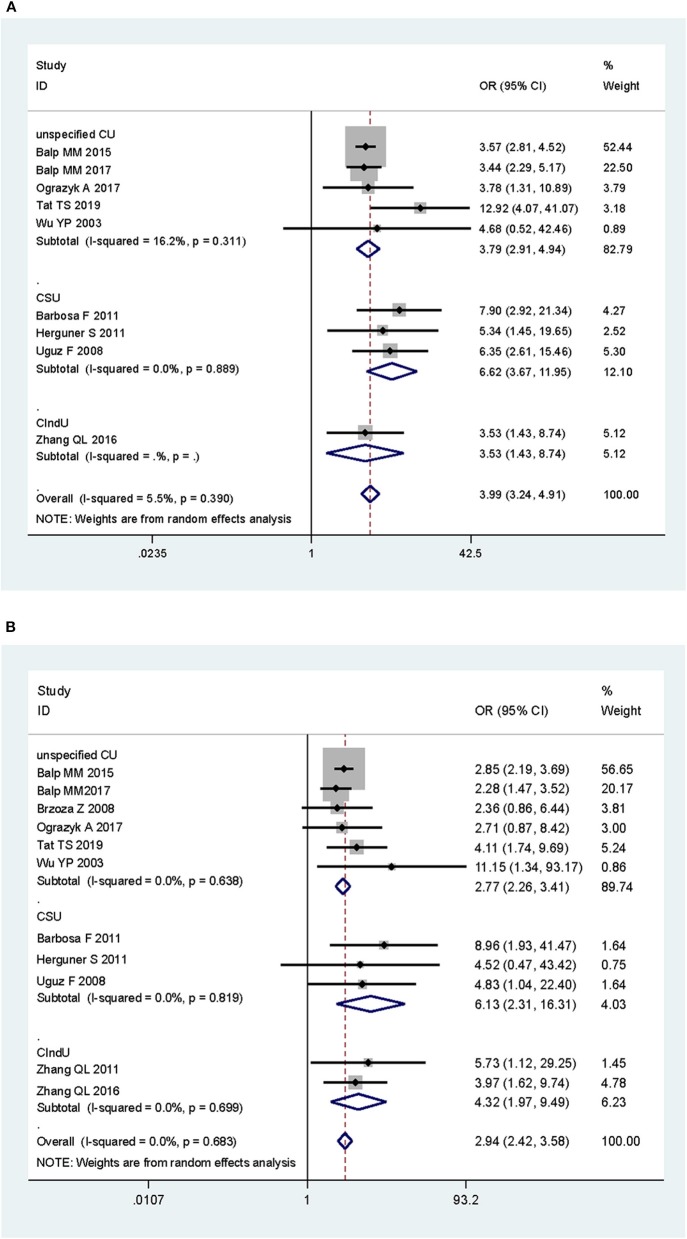
Subgroup analysis of the associations of chronic urticaria with symptoms of anxiety and depression in terms of odds ratio, by subtypes of chronic urticaria. **(A)** Anxiety and subtypes of chronic urticaria. **(B)** Depression and subtypes of chronic urticaria. CU, chronic urticaria; CSU, chronic spontaneous urticaria; CIndU, chronic-induced urticaria.

Subgroup analysis by disease subtypes is shown in Figure 3. The ORs of CSU, CIndU, and unspecified CU were 6.62 (95% CI: 3.67–11.95, *P* < 0.001), 3.53 (95% CI: 1.43–8.74, *P* < 0.001), and 3.79 (95% CI: 2.91–4.94, *P* < 0.001) for anxiety, respectively. The ORs of CSU, CIndU, and unspecified CU were 6.13 (95% CI: 2.31–16.31, *P* < 0.001), 4.32 (95% CI: 1.97–9.49, *P* < 0.001), and 2.77 (95% CI: 2.26–3.41, *P* < 0.001) for depression, respectively. As shown in Figure S1, the SMDs of CSU and unspecified CU were 1.15 (95% CI: 0.83–1.47, *P* < 0.001) and 0.82 (95% CI: 0.51–1.12, *P* < 0.001) for anxiety, and 0.99 (95% CI: 0.75–1.24, *P* < 0.001) and 0.61 (95% CI: 0.16–1.06, *P* = 0.001) for depression, respectively.

Subgroup analysis by tools of measurement is shown in Figure S2. The ORs varied from 3.53 (95% CI: 2.88–4.34, *P* < 0.001) to 7.11 (95% CI: 3.60–14.02, *P* < 0.001) for anxiety and from 2.36 (95% CI: 0.86–6.44, *P* < 0.001) to 4.85 (95% CI: 2.32–10.14, *P* < 0.001) for depression. The SMDs varied from 0.78 (95% CI: 0.27–1.28, *P* = 0.003) to 1.25 (95% CI: 0.77–1.72, *P* < 0.001) for anxiety and from 0.63 (95% CI: 0.19–1.07, *P* < 0.001) to 1.13 (95% CI: 0.66–1.60, *P* < 0.001) for depression. All pooled estimates were statistically significant.

### Sensitivity Analysis

**Figure 4:**
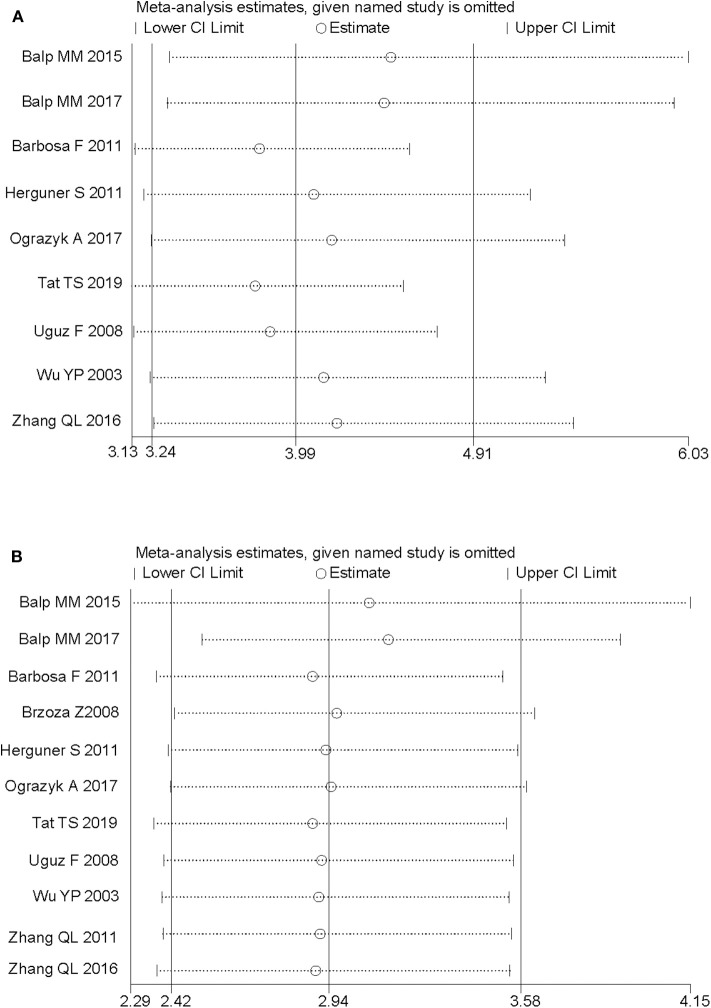
Sensitivity analysis of the pooled estimates by excluding the studies one by one. **(A)** Anxiety, in terms of odds ratio. **(B)** Depression, in terms of odds ratio.

We performed sensitivity analyses and assessed the relative impact of each study by excluding the studies one by one. The results showed no significant changes (Figure 4).

### Assessment on Quality of Studies

The risk of bias appraised among the included studies is shown in Table 2. Study quality scores ranged from 6 to 9, and all of them were of good quality.

**Table 2:** Quality assessment of the included studies.

**Study**	**Selection**				**Comparability**		**Exposure**			**Score**
	**1**	**2**	**3**	**4**	**5a**	**5b**	**6**	**7**	**8**	
Balp et al. (26)	√	√	√	√	√	√	×	√	√	8
Balp et al. (30)	√	√	√	√	√	×	×	√	√	7
Barbosa et al. (31)	√	×	√	√	√	√	√	√	×	7
Brzoza et al. (21)	√	×	√	√	√	×	√	√	√	7
Engin et al. (22)	√	√	√	√	√	√	√	√	√	9
Herguner et al. (24)	√	√	√	√	√	×	√	√	√	8
Ograzyk et al. (28)	√	×	√	√	√	×	×	√	√	6
Pasaoglu et al. (20)	√	√	√	√	√	√	√	√	√	9
Tat (29)	√	×	√	√	√	√	×	√	√	7
Uguz et al. (23)	√	√	√	√	√	√	√	√	√	9
Wu et al. (19)	√	√	√	√	√	√	√	√	√	9
Zhang and Hou (25)	√	×	√	√	√	√	√	√	√	8
Zhang et al. (27)	√	×	√	√	√	√	√	√	√	8

*1. Definition of case; 2. Representativeness of the cases; 3. Selection of controls; 4. Definition of controls; 5a. Study controls for age; 5b. Study controls for additional factor; 6. Ascertainment of exposure; 7. Same method of ascertainment for cases and controls; 8. Non-response rate*.

### Publication Bias

Egger's regression test (*P* = 0.050 for anxiety and *P* = 0.020 for depression) indicated significant publication bias, where the effect size (OR) was positively associated with study sample size.

## Discussion

In this meta-analysis, we analyzed 3,627 participants in 13 studies over the past 18 years, and investigated the associations of CU with the symptoms of anxiety and depression. We found that patients with CU had significantly higher risks and severities of anxiety and depression than health controls. Patients with CU had 3-fold risk of anxiety or depression, and patients with CSU had 6-fold risk of anxiety or depression, compared to the controls. The magnitude of associations varied moderately among the tools of measurement, but the conclusions remained consistent.

Many research studies support the association of mast cell activation with emotional problems. In a recent a study, depression levels in patients with mastocytosis were found to be significantly associated with tryptophan (TRP) levels (32). TRP metabolism changes in mastocytosis and is associated with stress and depression, suggesting that mast cells participate in the inflammatory pathway associated with depression. Mast cell activation also increases anxiety-like behavior (33), while anxiety and depression are associated with increased mast cell counts and degranulation (34). There is growing evidence that mast cells are critical for the pathogenesis of inflammatory diseases (32, 35). Studies show that depression can develop, in part, based on inflammatory changes, including interleukin-6 (IL-6), tumor necrosis factor-alpha (TNF-a), IL-1β, IL-2, and C-reactive protein (CRP) (36–38). In a meta-analysis of severe depressive cytokines, patients with depression had increased TNF-α and interleukin-6 levels compared to health controls (37). Therefore, mast cells play an important role in inflammation-induced depression (32, 39). We speculated that the relationship between CU and anxiety and depression was partly mediated by the release of inflammatory factors and the degranulation of mast cells.

In this meta-analysis, five studies included patients with CSU, three included CIndU, and five did not specify the subtype of CU. We observed a stronger correlation between CSU and anxiety/depression than CIndU or unspecified CU. A survey in 2013, however, reported that patients with inducible urticaria had more symptoms of anxiety and depression than those with CSU (40). Owing to the heterogeneities across included studies, it is difficult to conclude that the magnitude of association is larger in CSU than that in CIndU from this meta-analysis.

The measurement or diagnosis of anxiety and depression might serve as another important source of heterogeneity. In the subgroup analysis by the tool of measurement, the ORs varied from 3.53 to 7.11 for anxiety and from 2.36 to 54.85 for depression. The Hospital Anxiety and Depression Scale (HADS) has been widely used to assess the presence and severity of anxiety and depression in patients with physical illness (41). The HADS demonstrates high reliability with a Cronbach's alpha coefficient of 0.884 and test–retest correlation of 0.944 (42). However, in primary care settings, the HADS performs less well than the other instruments, such as the Beck Depression Inventory (43). Some authors suggest that the potential structure of the HADS in the sample is inconsistent and is highly dependent on the structure of establishing statistical methods (44, 45). The Self-Rating Anxiety Scale (SAS) developed by Zung has been widely used in the research and clinical practice of anxiety testing (46). The Cronbach's alpha of SAS is 0.897, and the test–retest reliability is 0.913 (47). The Zung Self-Rating Depression Scale (SDS) is designed to measure depression (48) and related symptoms with an alpha coefficient of 0.79 (49). However, some studies did not recommend to rely too much on the total score of SDS, since the various characteristics of heterogeneous symptoms are contained in a single dimension of severity (50, 51). In this meta-analysis, two studies by Balp et al. used a self-reported measure of anxiety and depression (26, 30), yet the reliability and validity were not mentioned by the authors. As a result, the variations in effect size were partly attributable to the tools of measurement.

The study has limitations. First, despite the strong association of CU with anxiety and depression, the causality could not be confirmed since all the included studies were cross-sectional or case-control studies. Eligible prospective cohort studies were not identified in the literature. A reversed causal relationship may exist, where CU might be triggered by changes in emotional status, possibly through mechanisms involving the hypothalamic–pituitary–adrenal axis or brain–gut–microbiome axis. Second, the variations in diagnosis and measurement of anxiety and depression may lead to substantial between-study heterogeneities. In addition, there was significant publication biases, which may be due to the fact that negative results were less likely to appear in published papers. The study also has strengths. First, this is the first meta-analysis and systematic review for the association of CU with symptoms of anxiety and depression. We confirmed the association through the synthesis of evidence, and the result has significant clinical implication for dermatologists. Second, we established strict inclusion and exclusion criteria, resulting in a relatively uniform data set. Including quality assessment was also a valuable asset of this assessment, allowing readers to judge the strength of evidence. In addition, research options and data extraction were completed by two independent reviewers, which help to ensure the comprehensiveness and accuracy of the review.

In conclusion, our present meta-analysis demonstrated that CU is associated with higher risks of anxiety and depression, and CSU might be associated with a larger effect on the risks.

## Data Availability Statement

All datasets generated for this study are included in the article/Supplementary Material.

## Author Contributions

YH, YX, and XZ searched the literature and extracted the data. YH and MS performed the systematic review, analyzed the data, and drafted the manuscript. JL and XC reviewed and revised the manuscript. XC obtained the funding. All authors gave final approval to the version submitted for publication.

### Conflict of Interest

The authors declare that the research was conducted in the absence of any commercial or financial relationships that could be construed as a potential conflict of interest.

## Supplementary Material

Figure S1 Subgroup analysis of the associations of chronic urticaria with symptoms of anxiety and depression in terms of standardized mean difference, by subtypes of chronic urticaria. **(A)** Anxiety and subtypes of chronic urticaria. **(B)** Depression and subtypes of chronic urticaria. CU, chronic urticaria; CSU, chronic spontaneous urticaria; CIndU, chronic-induced urticaria.

Figure S2 Subgroup analysis of the associations of chronic urticaria with symptom of anxiety and depression, by tool of measurement. **(A)** Tool of measurement by anxiety, in terms of odds ratio. **(B)** Tool of measurement by anxiety, in terms of standardized mean difference. **(C)** Tool of measurement by depression, in terms of odds ratio. **(D)** Tool of measurement by depression, in terms of standardized mean difference.
