# Supplementary figures and images for: A Meta-Analysis of Observational Studies on the Association of Chronic Urticaria With Symptoms of Depression and Anxiety
Source: Front Med (Lausanne). 2020 Feb 27;7:39. doi: 10.3389/fmed.2020.00039 (PMC7056669; doi:10.3389/fmed.2020.00039)

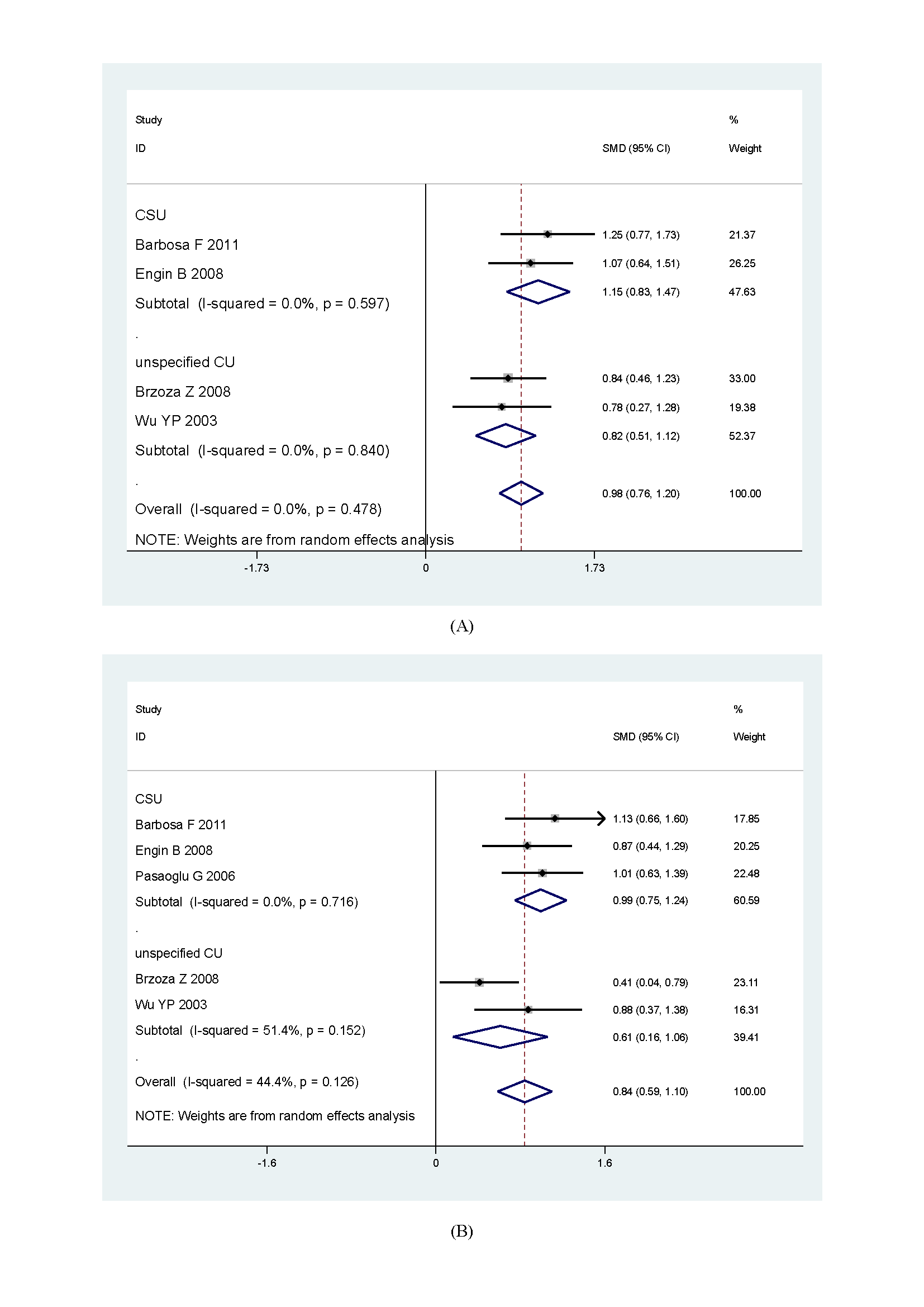

Supplement: Figure S1 — Subgroup analysis of the associations of chronic urticaria with symptoms of anxiety and depression in terms of standardized mean difference, by subtypes of chronic urticaria. (A) Anxiety and subtypes of chronic urticaria. (B) Depression and subtypes of chronic urticaria. CU, chronic urticaria; CSU, chronic spontaneous urticaria; CIndU, chronic-induced urticaria. [file Image_1.TIFF]

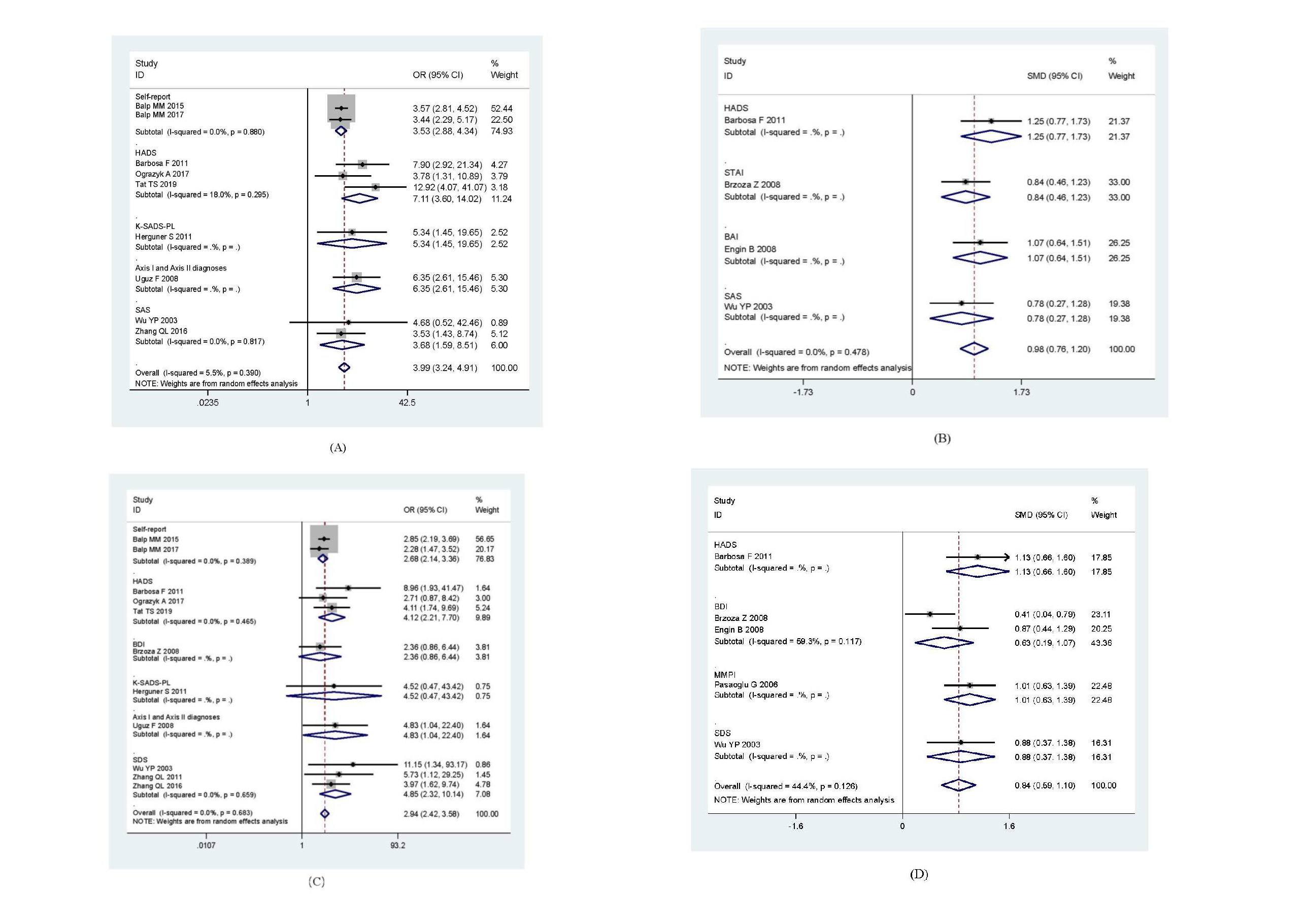

Supplement: Figure S2 — Subgroup analysis of the associations of chronic urticaria with symptom of anxiety and depression, by tool of measurement. (A) Tool of measurement by anxiety, in terms of odds ratio. (B) Tool of measurement by anxiety, in terms of standardized mean difference. (C) Tool of measurement by depression, in terms of odds ratio. (D) Tool of measurement by depression, in terms of standardized mean difference. [file Image_2.TIF]
